# Emblica Officinalis: A Novel Therapy for Acute Pancreatitis — An Experimental Study

**DOI:** 10.1155/1995/51310

**Published:** 1995

**Authors:** S. P. Thorat, N. N. Rege, A. S. Naik, U. M. Thatte, A. Joshi, K. N. S. Panicker, R. D. Bapat, S. A. Dahanukar

**Affiliations:** 1 Departments of Pharmacology Seth G. S. Medical College and K. E. M. Hospital Cancer Research Institute Tata Memorial Hospital, Parel Bombay 400 012 India; 2 Departments of Gastroenterology Surgery Seth G. S. Medical College and K. E. M. Hospital Cancer Research Institute Tata Memorial Hospital, Parel Bombay 400 012 India; 3 Departments of Pathology Seth G. S. Medical College and K. E. M. Hospital Cancer Research Institute Tata Memorial Hospital, Parel Bombay 400 012 India; 4 Cell Biology Division Cancer Research Institute Tata Memorial Hospital, Parel Bombay 400 012 India

## Abstract

Acute necrotising pancreatitis is associated with an unacceptably high mortality for which no satisfactory remedy exists. Emblica officinalis (E.o.) is a plant prescribed in Ayurveda, the Indian traditional system of medicine, for pancreas-related disorders. This study was carried out to evaluate the protective effect of E.o. against acute necrotising pancreatitis in dogs. Pancreatitis was induced by injecting a mixture of trypsin, bile and blood into the duodenal opening of the pancreatic duct. Twenty eight dogs were divided into 4 groups (n = 6-8 each): GpI–control, GpII–acute pancreatitis, GpIII–sham-operated, GpIV–pretreatment with 28 mg E.o./kg/day for 15 days before inducing pancreatitis. Serum amylase increased from 541.99 ± 129.13 IU/ml to 1592.63 ± 327.83 IU (p<0.02) 2 hrs after the induction of pancreatitis in GpII. The rise in serum amylase in both GpIII and GpIV was not significant. On light microscopic examination, acinar cell damage was less and the total inflammatory score was significantly lower in the E.o. treated group as compared to GpII. Electron microscopy confirmed this and showed an increased amount of smooth, endoplasmic reticulum and small, condensed granules embedded in a vacuole. More studies are needed to explore the clinical potential of E.o. and its mechanism of action.


*HPB Surgery*, 1995, Vol. 9, pp. 25–30
Reprints available directly from the publisher
Photocopying permitted by license only

© 1995 OPA (Overseas Publishers Association)
Amsterdam B.V. Published in The Netherlands
by Harwood Academic Publishers GmbH
Printed in Malaysia

### Emblica Officinalis: A Novel Therapy for Acute Pancreatitis—An Experimental Study

S. P. THORAT, N. N. REGE, A. S. NAIK*, U. M. THATTE, A. JOSHI**, K. N. S. PANICKER***, R. D. BAPAT* and S. A. DAHANUKAR

Departments of Pharmacology, *Gastroenterology Surgery and **Pathology, Seth G. S. Medical College and K. E. M. Hospital, ***Cell Biology Division, Cancer Research Institute, Tata Memorial Hospital, Parel, Bombay 400 012, India

*(Received October 23, 1993)*

Acute necrotising pancreatitis is associated with an unacceptably high mortality for which no satisfactory remedy exists. Emblica officinalis (E.o.) is a plant prescribed in Ayurveda, the Indian traditional system of medicine, for pancreas-related disorders. This study was carried out to evaluate the protective effect of E.o. against acute necrotising pancreatitis in dogs. Pancreatitis was induced by injecting a mixture of trypsin, bile and blood into the duodenal opening of the pancreatic duct. Twenty eight dogs were divided into 4 groups (n = 6–8 each): GpI—control, GpII—acute pancreatitis, GpIII—sham-operated, GpIV—pretreatment with 28 mg E.o./kg/day for 15 days before inducing pancreatitis. Serum amylase increased from 541.99 ± 129.13 IU/ml to 1592.63 ± 327.83 IU ($p < 0.02$) 2 hrs after the induction of pancreatitis in GpII. The rise in serum amylase in both GpIII and GpIV was not significant. On light microscopic examination, acinar cell damage was less and the total inflammatory score was significantly lower in the E.o. treated group as compared to GpII. Electron microscopy confirmed this and showed an increased amount of smooth endoplasmic reticulum and small, condensed granules embedded in a vacuole. More studies are needed to explore the clinical potential of E.o. and its mechanism of action.

KEY WORDS: Indian medicinal plant necrotising pancreatitis serum amylase electron microscopy of pancreas.

### INTRODUCTION

Acute pancreatitis usually results in a significant derangement of pancreatic function. Although most attacks are mild and subside without sequele in less than a week, the spectrum of this disease also includes necrotising pancreatitis which is associated with an unacceptably high morality rate of 79%[1]. The management of acute necrotising pancreatitis continues to pose therapeutic problems. No specific medical or surgical therapy is capable of directly limiting pancreatic autodigestion and inflammation, although various drugs, including $H_2$ blockers, anticholinergics, glucagon and aprotinin have been tried[2–4]. Treatment of the patient thus remains largely conservative, with attention directed towards maintaining an adequate circulatory volume, maximizing renal perfusion, supporting respiration and correcting electrolyte abnormalities.

Emblica officinalis is a medicinal plant, described in Ayurveda, the traditional medicinal system of India[5]. It is a moderate sized deciduous tree belonging to the Euphorbiaceae family. The fruit is the most commonly used part of this plant and is the richest natural source of vitamin C. The dried fruit has been prescribed in Ayurveda for pancreas-related disorders[6,7].

The present study was conceived to evaluate the effect of pretreatment with Emblica officinalis (E.o.) against experimentally induced acute necrotising pancreatitis in dogs.

*Address for correspondence:* Dr. Sadhna Thorat, A/8 Ramdarshan, Subhash Road, Vileparle (E), Bombay 400 057, India.

### METHODS AND MATERIALS

Twenty eight mongrel dogs of either sex were divided into 4 groups, each containing 6–8 animals as shown in Table 1.

**Table 1** Experimental Groups

| *Group* | *No. of animals* | *Procedure* |
|---|---|---|
| I | 6 | None |
| II | 8 | Induction of pancreatitis |
| III | 6 | Sham-operation |
| IV | 8 | 15 days oral pretreatment with Emblica officinalis in the dose of 28 mg/kg/day followed by induction of pancreatitis on day 16. |

### Induction of Pancreatitis

The dogs were anaesthetised with pentobarbitone sodium. An upper midline abdominal incision was taken. The peritoneum was opened and after incising the second part of the duodenum, the papilla of the main pancreatic duct was identified. Acute pancreatitis was induced by injecting 10 ml of a mixture of trypsin, blood and bile into the duodenal opening of the main pancreatic duct as described by Wright and Goodhead[8]. One gram trypsin powder (obtained from Fluka Chemie AG, Switzerland) was dissolved in 5 ml normal saline. To this were added 2 ml bile, aspirated from the gall bladder, using a guage 20 needle and 3 ml blood, obtained from the cannulated femoral vein, both from the same dog.

Group III i.e. the sham-operated group was included to rule out the effect of stress of surgery and anaesthesia on the parameters used in the study. In dogs belonging to this group, the duodenum was opened, the pancreatic duct opening visualized and duodenum closed.

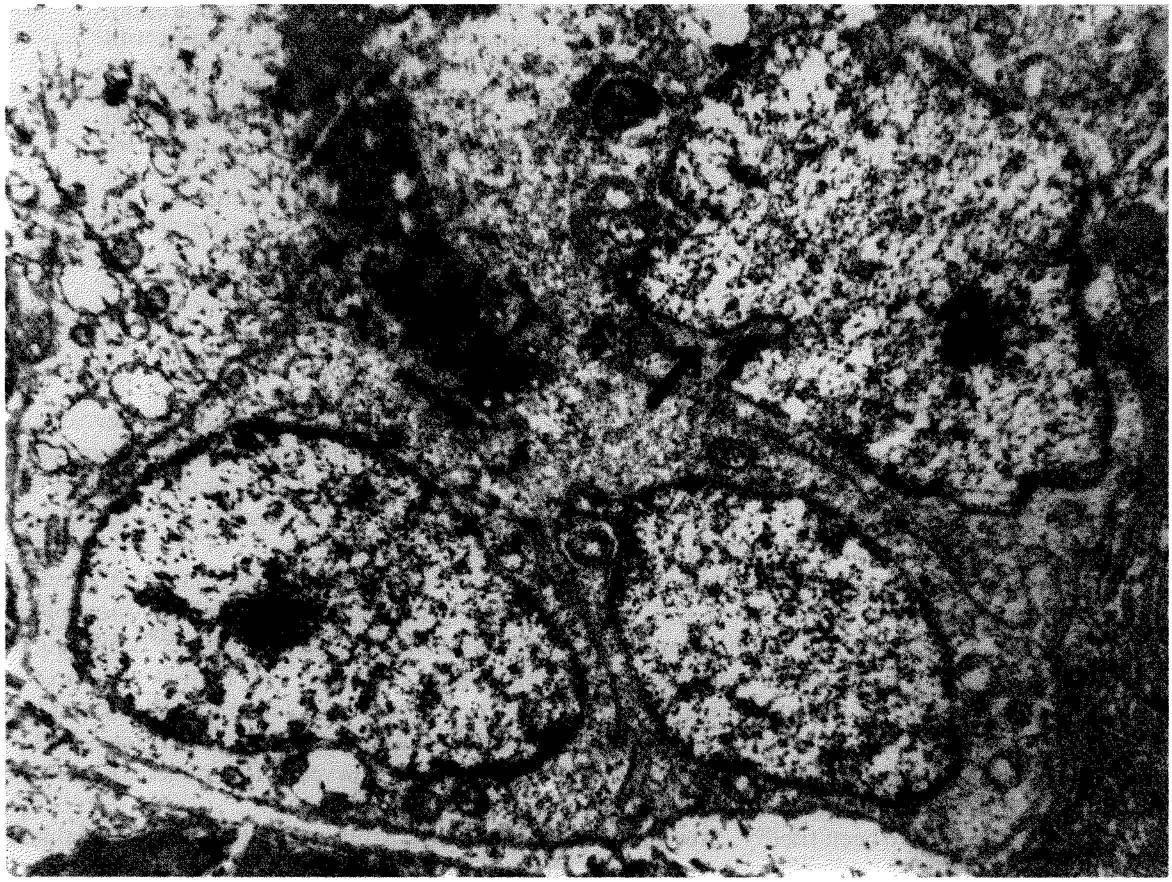

**Figure 1** Photoelectronmicrograph of the pancreas 2 hrs after the induction of pancreatitis. Note the irregular, crenated nucleus (↑) with altered nucleo-cytoplasmic ratio (× 14,700).

### Drug Administration

A fine powder of the dried fruit of E.o. was administered to the animals in group IV in the dose of 28 mg/kg/day for 15 days as part of their regular diet.

### Assessment of Drug Effect

The following parameters were monitored during the study. *Serum amylase* was measured based on the modified saccharogenic method using a commercially available kit, before and 2 hrs after the induction of pancreatitis or sham-operation.

*Light microscopic examination* of a pancreatic biopsy specimen was done at 2 hrs after the induction of pancreatitis. All the biopsy specimens were coded and sent for histopathological examination. Depending on the extent of necrosis, haemorrhage and edema, each was graded as 0-absent, 1-mild, 2-moderate, 3-severe. Polymorphonuclear infiltration was graded as 0-absent and 1-present. The total inflammatory score of all the 4 scores (i.e. that for necrosis, haemorrhage, edema and polymorphonuclear infiltration) was considered for comparison between the 4 groups (minimum score-0, maximum score-10).

*Electron microscopic (EM) examination* of the pancreatic biopsy specimen taken 2 hrs after the induction of pancreatitis was carried out. The tissue blocks were preserved in sodium cacodylate buffer and cut on a LKB 2088 ultramicrotome. They were then picked up on copper grids. The grids were viewed under a Zeiss EM 109 electron microscope.

Statistical analysis was performed on the parametric data by applying students 't' test and on non-parametric data using Mann Whitney test.

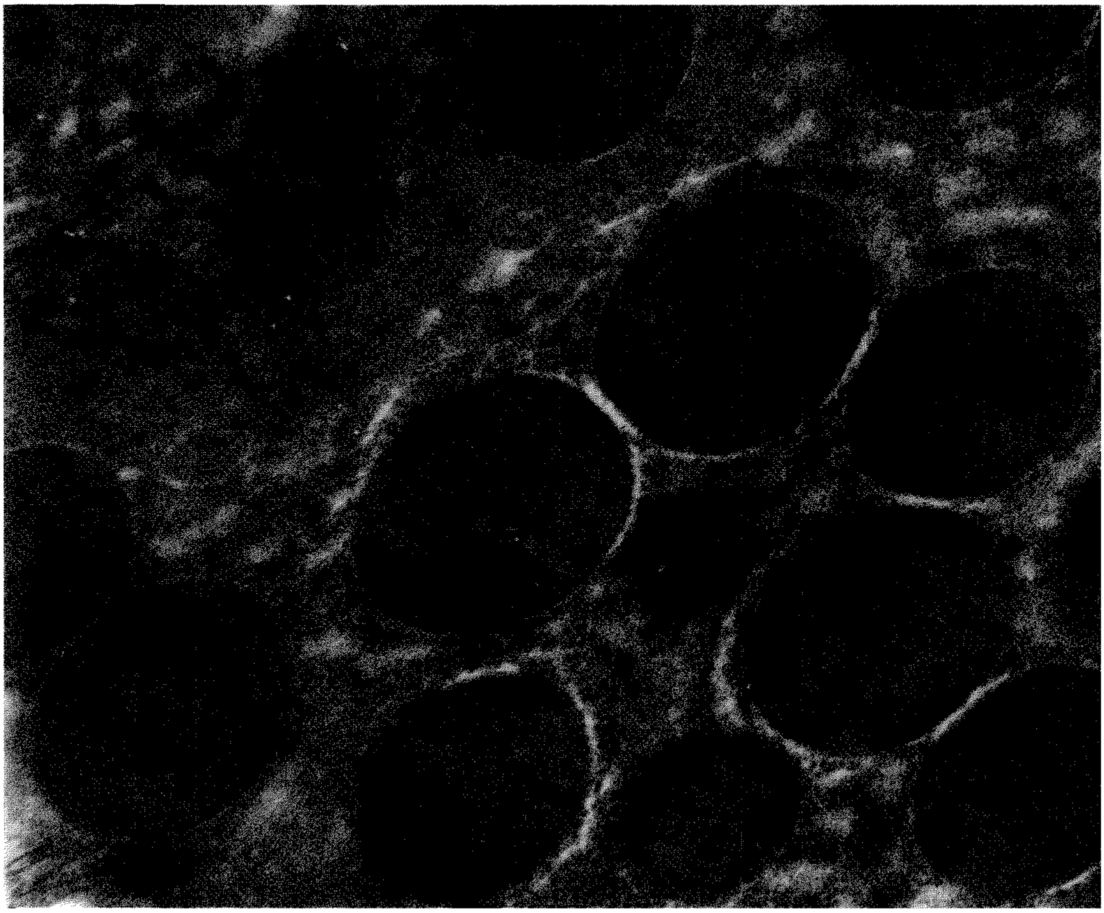

**Figure 2** Photoelectronmicrograph of the pancreas showing semi-electron dense, large oval granules in the cytoplasm (× 36,000).

### RESULTS

The serum amylase (Mean ± SE) was 506.65 ± 26.16 IU/ml in normal dogs at O hr and 525.17 ± 18.3 IU/ml at 2 hrs. After the induction of pancreatitis in group II, the serum amylase increased from 541.99 ± 129.13 IU/ml to 1592.63 ± 327.83 IU/ml at the end of 2 hrs ($p < 0.02$). Sham-operated animals (group III) showed a non-significant rise in serum amylase from 587.52 ± 71.63 IU/ml to 701.31 ± 60.45 IU/ml at 2 hrs. In group IV, although serum amylase increased from 588.36 ± 120.97 IU/ml to 1043.77 ± 374.45 IU/ml, this rise was not statistically significant. Further, the serum amylase at 2 hrs in group IV was significantly less as compared to that in group II at 2 hrs ($p < 0.05$).

The pancreatic biopsies taken from group I showed normal pancreatic architecture. Therefore the total inflammatory score was 0. Area of haemorrhage and necrosis (grade 3 each) were seen to replace normal acinar cells in group II. This was associated with edema of remaining acinar cells (grade 3) and polymorphonuclear infiltration (grade 1). The total inflammatory score of this group was 10. The pancreas of 3 dogs in the sham-operated group showed mild edema while the rest were normal. The average total inflammatory score in this group was 0.66 ± 0.81. In Group IV, the degree of architectural damage (haemorrhage, necrosis, edema) was significantly reduced when compared with that in group II. There was no evidence of polymorphonuclear infiltration and the mean total inflammatory score was 2.143 ± 1.46 ($p < 0.05$).

Electron microscopic examination of the pancreas from group II revealed cells with highly irregular, crenated nuclei and marginal condensation of the chromatin material. The nucleo-cytoplasmic ratio was altered (Figure 1). A large number of semi-electron dense, large oval granules was seen in the cytoplasm (Figure 2). The EM examination in group IV showed

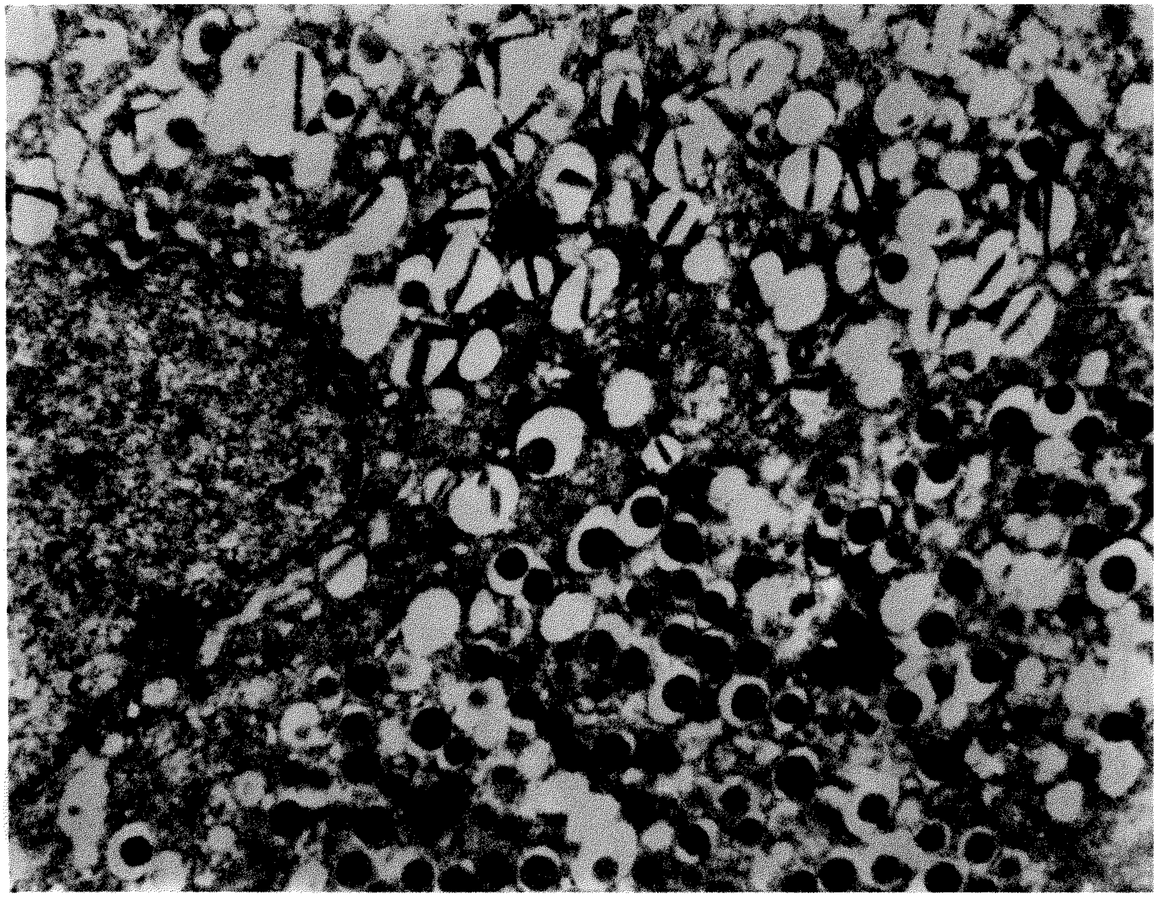

**Figure 3** Photoelectronmicrograph of the pancreas from GpIV (pretreated with E.o.) showing small granules embedded in vacuoles (× 21, 600).

cells with indented nuclei. However, in contrast to the picture in group II, many cells showed a large number of small uniform, electron-dense granules (Figure 3). The membrane of zymogen granules appeared intact. Further, smooth endoplasmic reticulum was seen to be increased in these cells as compared to cells in group II (Figure 4).

### DISCUSSION

This study showed for the first time the potential of Emblica officinalis in preventing experimental acute pancreatitis.

Acute pancreatitis continues to represent a vexing clinical entity. The pathobiology of the disease process is ill understood and few effective therapies exist. Therefore, the management of acute pancreatitis has been mainly supportive and non-specific. In this study, we have evaluated the efficacy of E.o. in a model of acute necrotising pancreatitis in dogs.

Intraductal injection of a mixture of trypsin, blood and bile induces acute necrotising pancreatitis by virtue of the detergent and toxic effects of bile on the acinar cells and activation of the proteolytic enzymes, leading to autodigestion[9]. This was confirmed in our study both biochemically (statistically significant rise in serum amylase) and histopathologically in group II. The rise in serum amylase in the sham-operated dogs was not significant. Also, mild edema was seen on histopathology in only 3 out of 8 dogs in this group and the total inflammatory score was significantly lower than that of group II. These changes, suggestive of minimal damage to the pancreas could be due to the handling of the pancreas. Further, they proved that the findings in group II were due to pancreatitis itself and not due to the stress of surgery or anaesthesia. When pancreatitis was induced in group IV, serum

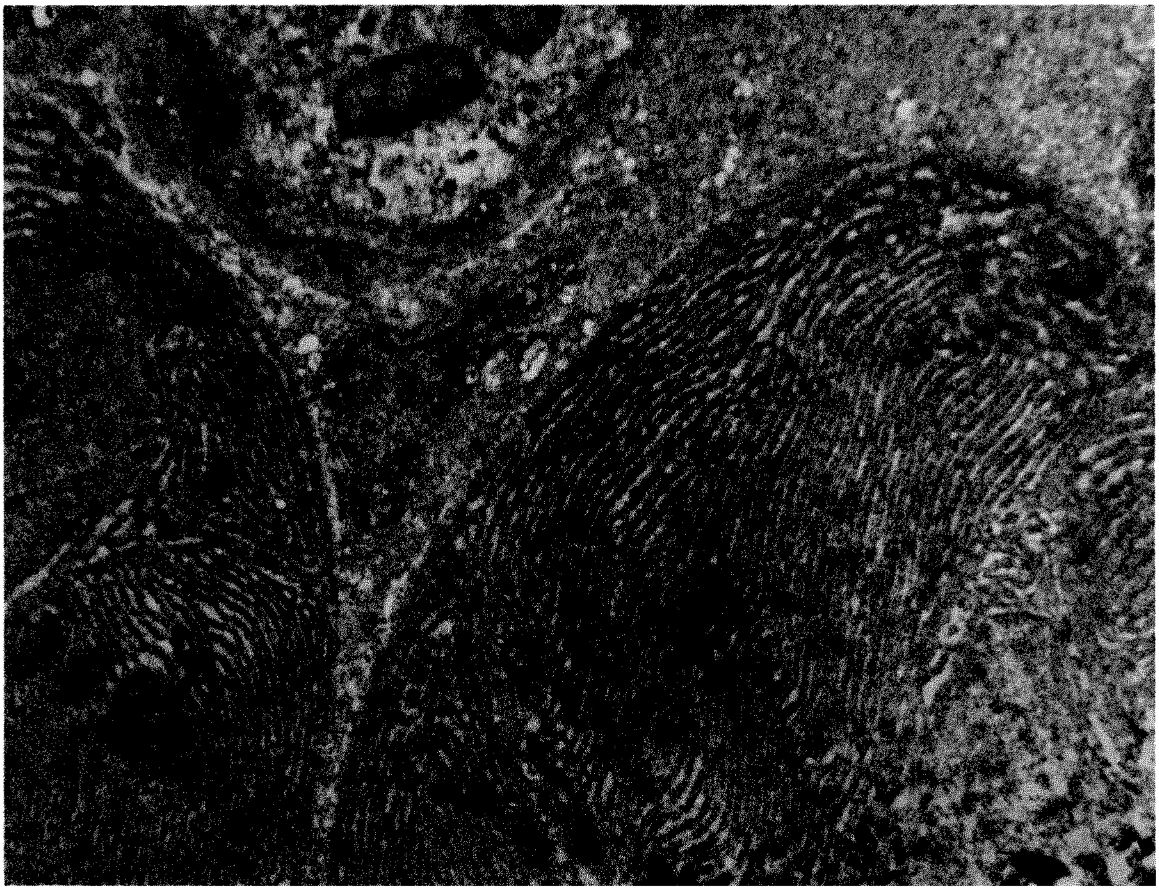

**Figure 4** Photoelectronmicrograph of the pancreas from GpIV showing proliferation of the smooth endoplasmic reticulum (↑) (× 6,930).

amylase increased 2 hrs after the procedure. This rise was however not statistically significant. Light microscopic examination of pancreas at 2 hrs showed that the pancreatic cells were slightly edematous. No foci of haemorrhage or necrosis were observed. It has already been reported that an injection of saline or even air under pressure can cause edematous pancreatitis and a rise in serum amylase[10]. This can explain the rise in serum amylase as well as the edematous appearance of the pancreas in the E.o. treated animals.

The electron microscopic examination corroborated the findings of histopathology. Steer and Meldolesi[11] have discussed the cell biology of experimental pancreatitis. They suggest that the critical event in pancreatitis occurs within the acinar cells due to deranged transport or secretion. Lysosomal enzymes such as cathepsin B may be responsible for the intracellular activation of digestive enzymes. Therefore, treatments aimed at restoring the normal patterns of intracellular transport and secretion, as well as preventing activation of digestive enzymes by lysosomal hydrolases might prove beneficial in the management or prevention of pancreatitis. The EM finding in E.o. treated dogs suggests that E.o. could have prevented the fusion of lysosomes and zymogen granules, thereby preventing the activation of digestive enzymes. Another remarkable feature of the EM examination of the pancreas of dogs from group IV was the proliferation of smooth endoplasmic reticulum, the significance of which is not known. The physiological function of smooth endoplasmic reticulum is to synthesize phospholipids and triglycerides. Thus, an increased synthesis of phospholipids and triglycerides could have contributed to an increased defence of the acinar cells against the trypsin-blood-bile onslaught.

This experimental study showed conclusively that pretreatment with Emblica officinalis prevents acute necrotising pancreatitis. Further studies to look into the exact mechanism of its protective action are needed. Moreover, the clinical potential of this plant in acute on chronic pancreatitis needs to be explored.

### REFERENCES

1. Seligson, U. (1982) Diagnostic criteria, classification, clinical course in pancreatitis. *Acta Chirugie Scandinavia*, **512** (Suppl), 1.
2. Loiudice, T. A., Land, J. and Mehta, J. (1984) Treatment of acute alcoholic pancreatitis—the roles of cimetidine and nasogastric suction. *American Journal of Gastroenterology*, **79**, 553–558.
3. Soergel, K. H. (1978) Medical treatment of acute pancreatitis-what is the evidence? *Gastroenterology*, **74**, 620.
4. Medical Research Council Multicentre Trial. (1980) Morbidity of acute pancreatitis—The effect of aprotinin and glucagon. *Gut*, **21**, 334–339.
5. Gogate, V. M. (1982) Ayurvedic Materia Medica, 1st edn, pp. 247–248. Bombay: Continental Prakashan.
6. Garde, G. K. (1970) Sarth Vagbhat, 6th edn, p. 40 (Sutra 40). Pune: Aryabhushana Mudranalaya.
7. Gogate, V. M. (1982) Ayurvedic Materia Medica, 1st edn, p. 675. Bombay: Continental Prakashan.
8. Wright, P. W. and Goodhead, B. (1970) Prevention of haemorrhagic pancreatitis with fibrinolysin or heparin. *Archives of Surgery*, 100, 42–46.
9. Creutzfeldt, W., Lankisch, P. G. (1985) Acute pancreatitis: Etiology and pathogenesis. In Bockus Gastroenterology, edited by J. E. Berk, 4th edn, pp. 3971–3992. Philadelphia: WB Saunder Co.
10. Satake, K., Chung, Y. S., Yoshimoto, T., Ohyama, T. and Katayama, K. (1982) Radioimmunoreactive serum elastase levels and histologic changes during experimental pancreatitis. *Archives of Surgery*, **117**, 777–780.
11. Steer, J. S. and Meldolesi, J. (1987) The cell biology of experimental pancreatitis. *New England Journal of Medicine*, **316**, 144–150.

